# Long-term habitat changes in a protected area: Implications for herpetofauna habitat management and restoration

**DOI:** 10.1371/journal.pone.0192134

**Published:** 2018-02-14

**Authors:** Chantel E. Markle, Gillian Chow-Fraser, Patricia Chow-Fraser

**Affiliations:** Department of Biology, McMaster University, Hamilton, Ontario, Canada; University of South Carolina, UNITED STATES

## Abstract

Point Pelee National Park, located at the southern-most tip of Canada’s mainland, historically supported a large number of herpetofauna species; however, despite nearly a century of protection, six snake and five amphibian species have disappeared, and remaining species-at-risk populations are thought to be in decline. We hypothesized that long-term changes in availability and distribution of critical habitat types may have contributed to the disappearance of herpetofauna. To track habitat changes we used aerial image data spanning 85 years (1931–2015) and manually digitized and classified image data using a standardized framework. Change-detection analyses were used to evaluate the relative importance of proportionate loss and fragmentation of 17 habitat types. Marsh habitat diversity and aquatic connectivity has declined since 1931. The marsh matrix transitioned from a graminoid and forb shallow marsh interspersed with water to a cattail dominated marsh, altering critical breeding, foraging, and overwintering habitat. Reduced diversity of marsh habitats appears to be linked to the expansion of invasive *Phragmites australis*, which invaded prior to 2000. Loss of open habitats such as savanna and meadow has reduced availability of high quality thermoregulation habitat for reptiles. Restoration of the northwestern region and tip of Point Pelee National Park to a mixed landscape of shallow wetlands (cattail, graminoid, forb, open water) and eradication of dense *Phragmites* stands should improve habitat diversity. Our results suggest that long-term landscape changes resulting from habitat succession and invasive species can negatively affect habitat suitability for herpetofauna and protection of land alone does not necessarily equate to protection of sensitive herpetofauna.

## Introduction

The rate of landscape change and the extent at which it affects species-at-risk has escalated since the onset of the Anthropocene [1]. Many ecosystems have become increasingly composed of a mix of historical and novel habitats, making it difficult to evaluate future conservation actions and resource management strategies [2]. However, this decision-making process benefits from understanding the historic landscape and tracking long-term environmental changes [3,4]. Identification of long-term trends in habitat changes may elucidate drivers of ecological processes, and therefore, help understand mechanisms of landscape changes [5]. This is important because, while habitat succession is a natural process, succession and changes can affect landscape suitability either positively or negatively depending on the target species.

Landscape change through fragmentation and the direct loss of habitat prohibits or significantly impairs species’ ability to carry out critical life activities such as foraging, overwintering, and breeding. In addition, habitat loss and fragmentation can isolate critical habitats or entire populations (e.g., [6]), reduce genetic diversity (e.g., [7]), decrease the potential for rescue effects (e.g., [8]), and result in local species extinctions [9,10]. Reptiles and amphibians, collectively referred to as herpetofauna, have experienced recent global population declines frequently linked to habitat loss, fragmentation, and degradation [11–15]. Herpetofauna depend on diverse wetland habitat for breeding, foraging, and winter refugia. In addition to wetlands, many reptiles and amphibians rely on adjacent terrestrial habitat [16]. For instance, turtles require terrestrial habitat for nesting and estivation [17,18], some snakes hibernate on land [19,20], and after breeding, some anurans will move upland to forage and overwinter [21]. A tight spatial coupling between wetland and terrestrial habitat is critical for a diverse range of herpetofauna to successfully carry out life activities.

The protection of natural lands is vital for conservation of biodiversity [22]; however, protection of lands alone is not always sufficient for maintaining historic species diversity. This seems to be the case for Point Pelee National Park (PPNP) located at the southern-most tip of Canada’s mainland, which has lost several herpetofauna species despite nearly a century of protection from human disturbance [23]. Although agricultural practice and human infrastructure has been limited within the protected park, there have been suspected changes in appropriate habitat for herpetofauna. Over time, the park has seen the extirpation of five amphibian (i.e., Northern cricket frog (*Acris crepitans*), Eastern tiger salamander (*Ambystoma tigrinum*), Fowler’s toad (*Anaxyrus fowleri*), gray treefrog (*Hyla versicolor*), American bullfrog (*Lithobates catesbeianus*)) and six snake species (i.e., blue racer (*Coluber constrictor foxii*), timber rattlesnake (*Crotalus horridus*), gray ratsnake (*Pantherophis spiloides*), Eastern hog-nosed snake (*Heterodon platirhinos*), milksnake (*Lampropeltis triangulum*), massasauga (*Sistrurus catenatus*)), with a suspected extirpation of the Endangered spotted turtle (*Clemmys guttata*) that was last seen in the park in the early 1990s [24]. Currently, the park supports several at-risk reptile species, though evidence suggests that recruitment is limited for the snapping turtle population (*Chelydra serpentina* [25]). Continued loss of herpetofauna is of conservation concern, and identification and quantification of habitat changes in the park can inform management and habitat restoration efforts.

Our primary objective was to determine if habitat changes have occurred within PPNP over the last 85 years, and subsequently, to quantify changes in habitat area, type and configuration. Given that PPNP has been protected from anthropogenic stressors such as habitat alteration, long-term changes in availability and distribution of habitats types may have contributed to the disappearance of herpetofauna. We use a multi-scale approach to examine changes in habitat critical for herpetofauna. Specifically, we evaluate spatiotemporal changes in shallow marsh habitats suitable for breeding, feeding and overwintering for turtles [18] and anurans [21], upland and sandy habitats that provide nesting habitat for turtles [18], and thermoregulation habitat and refugia for snakes [26,27]. Our second objective was to identify areas that have experienced the greatest habitat changes or loss of suitable herpetofauna habitat.

## Methods

### Study site

Point Pelee is a 16 km^2^ National Park located on the north shore of Lake Erie at the southernmost tip of Canada’s mainland. The park was established in 1918 and is a popular tourist destination, hosting 300,000–500,000 visitors annually [23]. Point Pelee National Park is located within the Carolinian zone, one of the most diverse Canadian regions for herpetofauna. Point Pelee is also recognized as an Important Bird Area and a Wetland of International Significance by UNESCO [28].

### Habitat delineation and classification

Historical aerial photography is an effective way of informing ecological restoration surrounding herpetofauna, because it captures spatial and temporal changes in herpetofauna critical habitat suspected to drive these species’ declines [11,13]. Unlike other historical records, repeat aerial imagery present the opportunity to help guide justifiable restoration goals by assessing environmental baselines and the variability of ecological processes over time [5]. Moreover, it allows for measuring changes in spatial patterns of critical habitat important to species of interest (e.g., [29,30]). To quantify habitat changes that have occurred in PPNP, we acquired image data for the years 1931, 1959, 1973, 1977, 1985, 1990, 2000, 2004, 2010 and 2015 to complete a multi-date data classification (Table 1). We digitized image data and classified habitats in ArcGIS 10.3 (ESRI, Redlands, California, USA) at a map scale of 1:1500. When examining habitat change derived from image data, our detection analyses are only as accurate as each individual classified product [31]. These errors are inherent with any historical image classification since derived data cannot be ground-truthed. We minimized compound errors by creating and adhering to a formal delineation and classification framework for each image (Table 2; S1 Table). To maintain nomenclature consistency, we adapted habitat ecosite and vegetation names from Dougan and Associates [32], who previously completed an Ecological Land Classification for Point Pelee National Park. Although we classified the delineated habitats to ecosite, not all ecosite classes were used to facilitate comparisons among years. Therefore, agriculture, constructed, forest, meadow, woodland, savanna, thicket and swamp were used at the community class level in our final analyses (Table 2).

**Table 1:** Image data processed for change-detection analyses in Point Pelee National Park (PPNP).

Year	Source	Date of Acquisition	Season	Spectral Range	Scale
1931	Air photo; obtained from PPNP	4/18/1931	Leaf off	Black and White	1:10,000
1959	Air photo; obtained from PPNP	4/29/1959	Leaf off	Black and White	1:4,000
1973	Air photo; obtained from PPNP	1/13/1973	Leaf off	Black and White	1:10,000
1977	Air photo; obtained from PPNP	11/2/1977	Leaf off	Black and White	1:4,000
1985	Air photo; obtained from PPNP	Summer 1985	Leaf on	Black and White	1:30,300
1990	Air photo; obtained from PPNP	4/8/1990	Leaf off	Black and White	1:9,000
2000	Air photo; obtained from PPNP	3/22/2000	Leaf off	Black and White	1:6,250
2004	Air photo; obtained from PPNP	4/9/2004	Leaf off	Black and White	1:10,000
2010	Land Information Ontario; Southwestern Ontario Orthophotography Project (SWOOP)	Spring 2010	Leaf off	True Colour	1:10,000
2015	Land Information Ontario; Southwestern Ontario Orthophotography Project (SWOOP)	Spring 2015	Leaf off	True Colour	1:10,000

**Table 2:** Habitat types classified in image data for Point Pelee National Park.

Habitat Class	Description
Agriculture	Active or inactive agricultural fields, including coniferous plantations and orchards.
Constructed	Buildings, roads and trails.
Common Reed Graminoid MMM	Dense invasive *Phragmites australis australis* typically growing in circular shapes throughout the marsh.
Cattail OSM	Very low edge-to-interior ratio (high density) of cattails (T. *latifolia*, *T*. *angustifolia*, *T*. *x glauca*) with homogenous appearance, almost no visible pools of open water.
Graminoid OSM	Medium edge-to-interior ratio of mixed broad-leaved or narrow-leaved emergents interspersed with water (medium density).
Forb OSM	High edge-to-interior ratio of broad-leaved or narrow-leaved emergents with many small pools (low density).
Graminoid MMM	Little to no standing water, dense monospecific grass stands.
Mixed MMM	Small, wet meadow with diverse floral communities, often found between dune and extensive marsh communities.
Open Water	Open water, devoid of vegetation
Meadow	Open terrestrial area with homogenous spread of grasses, < 25% tall shrub cover. Includes dry-fresh graminoid and mixed meadow ecosites.
Forest	> 75% cover of deciduous or coniferous, or > 25% both coniferous and deciduous. Includes dry-fresh cedar, poplar, deciduous, white pine forest, and fresh-moist poplar forest ecosites.
Woodland	35–60% cover of deciduous or coniferous trees. Includes dry-fresh cedar coniferous, deciduous and mixed woodland ecosites.
Savanna	10–35% cover of deciduous or coniferous trees. Includes dry-fresh cedar coniferous and mixed savanna ecosites.
Thicket	Open area with > 25% tall shrub cover and little tree cover. Includes dry-fresh and fresh-moist deciduous thicket, and deciduous shrub thicket ecosites.
Swamp	Wetland characterized by presence of deciduous or coniferous trees. Includes maple mineral deciduous swamp, and mineral deciduous thicket swamp ecosites.
Sand Barren/ Dune	Low hill or ridge of sand either inland or lining the land, vegetation may include shrubs and trees. Includes open, shrub, treed sand barren and dune ecosites.
Shoreline	Community that lies adjacent to Lake Erie, rarely any vegetation. Includes mineral open and shrub shoreline ecosites.

### Habitat loss and fragmentation

We measured habitat areas in ArcGIS 10.3 and calculated change in total area on a temporal scale. We expressed all habitat types as a percentage of the total delineated park area and compared relative changes in habitat losses and gains over time. Though marsh water levels may have fluctuated between years, underlying substrate generally maintains habitat type and instead would result in a change in vegetation density. Total area of park should, therefore, be more representative of temporal variation in habitat composition. To determine if serial autocorrelation was present in our dataset, we used the Durbin-Watson test. We also used a spearman’s correlation to determine if change in habitat area was correlated with Lake Erie water levels (Data obtained from Great Lakes Water Level Dashboard, www.glerl.noaa.gov).

We calculated a variety of metrics in FragStats 4.2 [33] to quantify habitat loss and fragmentation within the park at both the landscape and habitat class scale. For the purposes of our study, we defined the landscape as Point Pelee National Park, comprising a mosaic of habitat patches. At the landscape scale, we investigated changes to the area and distribution of patches, as well as overall habitat diversity using Shannon’s and Simpson’s diversity index and Simpson’s evenness. At the class scale, we analyzed changes to the area and distribution of specific habitat classes. We used linear regression to determine the relationship between fragmentation metrics and time. Prior to calculating metrics, we converted habitat data to rasters with a cell size of 2 m to ensure patch boundaries were accurately represented and that visually connected features remained connected in the raster surface. To characterize fragmentation, we quantified change in landscape division and patch distribution using effective mesh size and splitting index [33]. The splitting index is the number of patches after dividing the landscape into patches of equal size that would result in the same degree of landscape division, and is irrespective of patch size, shape, and relative location. Effective mesh size is the size of the patches when the landscape is divided in S areas of the same size (where S = splitting index).

We quantified habitat change using patch and core area metrics standardized by the proportional abundance of each habitat type (area-weighted metrics [33]). We calculated mean patch area and the mean radius of gyration to measure patch extent and to determine the average distance a species can move within a specific patch before reaching the patch boundary. Lastly, we calculated core area metrics to estimate the amount of interior habitat after accounting for edge habitat. We quantified the change in core area, number of disjunct core areas, and the core area index (i.e., the percentage of core area). Examining core area is important because the measure integrates both the size and shape of the patch. The amount of core area will drastically differ between two patches of the same total size if one patch is a perfect circle and the second is elongated and narrow. Core area metrics provide an indication of how far a species would have to travel before accessing another habitat type, as well as the ratio of edge to interior habitat, a variable that is relevant to species adversely affected by edges [34] or that select for edge habitat [35,36]. When determining core area, we treated 15 m as edge habitat based on the average daily distance moved by spotted turtles (30 m [37,38]) and previous research on habitat use by snakes [35,36].

### Change-detection

To identify change hotspots that could be used to direct restoration, we conducted a change-detection analysis in ArcGIS 10.3. We examined change-detection rasters to identify which habitat types replaced those that had been lost. We chose images that had been acquired many decades apart. We could not use the earliest image acquired in 1931 due to low resolution, but instead used the 1959 image, which had a suitably high resolution, and the 2015 image, which is the most recently available.

## Results

### Landscape-level habitat changes

At the landscape scale, habitat in Point Pelee National Park has become less diverse and more homogenous. In all cases the Durbin-Watson test was not significant (DW < 2, *p* > 0.5); therefore, no serial autocorrelation was present in our dataset. Mean patch area and mean patch extent (radius of gyration) increased at a rate of 1.84 ha per year and 2.98 ha per year, respectively (Table 3). The amount of mean core habitat in PPNP increased by 200% from 1931 to 2015 (Table 3). The mosaic of unique natural habitats declined through time from 26 to 8 patches (splitting index) and, on average, each patch increased by 128 ha (effective mesh size; Table 3).

**Table 3:** Metrics used to describe habitat changes in Point Pelee National Park at the landscape and class scale.

Scale	Habitat	Metric	R^2^	Rate of change	*p* -value	1931	2015
Landscape	n/a	AW Mean Patch Area	0.81	1.84 ha/yr	0.0009	67.7 ha	186.0 ha
		AW Mean Radius of Gyration	0.67	2.98 m/yr	0.0066	532.1 m	714.1 m
		AW Mean Core Area	0.82	1.64 ha/yr	0.0008	50.8 ha	155.0 ha
		Effective Mesh Size	0.84	1.92 ha/yr	0.0005	58.4 ha	186 ha
		Splitting Index	0.91	-0.23 patches/yr	< 0.0001	26.4 patches	8.0 patches
		Shannon’s Diversity Index	0.92	-0.008 /yr	< 0.0001	2.4	1.8
		Simpson’s Diversity Index	0.90	-0.002 /yr	0.0001	0.88	0.75
		Simpson’s Evenness	0.89	-0.002 /yr	0.0001	0.93	0.80
Class	Cattail OSM	AW Mean Patch Area	0.84	4.12 ha/yr	0.0005	63.9 ha	336.1 ha
		AW Mean Radius of Gyration	0.82	7.43 m/yr	0.0007	377.4 m	903.2 m
		Effective Mesh Size	0.83	2.12 ha/yr	0.0006	12.8 ha	141.8 ha
	Open Water	AW Mean Core Area Index	0.57	0.11%/yr	0.01	75.1%	83.4%
		Number of Disjunct Core Areas	0.55	-0.73 areas/yr	0.01	79	33
	Forb OSM	AW Mean Core Area Index	0.57	-0.53%/yr	0.02	49.6%	0.00%
	Forest	AW Mean Patch Area	0.82	0.47 ha/yr	0.0004	4.2 ha	49.8 ha
		AW Mean Radius of Gyration	0.75	4.68 m/yr	0.0012	128.3 m	558.8 m
	Meadow	AW Mean Core Area Index	0.46	-0.46	0.03	64.1%	33.7%

Each metric was regressed against year to determine rate of change during our study period (1931–2015). AW = area-weighted.

### Class-level habitat changes

**Fig 1:**
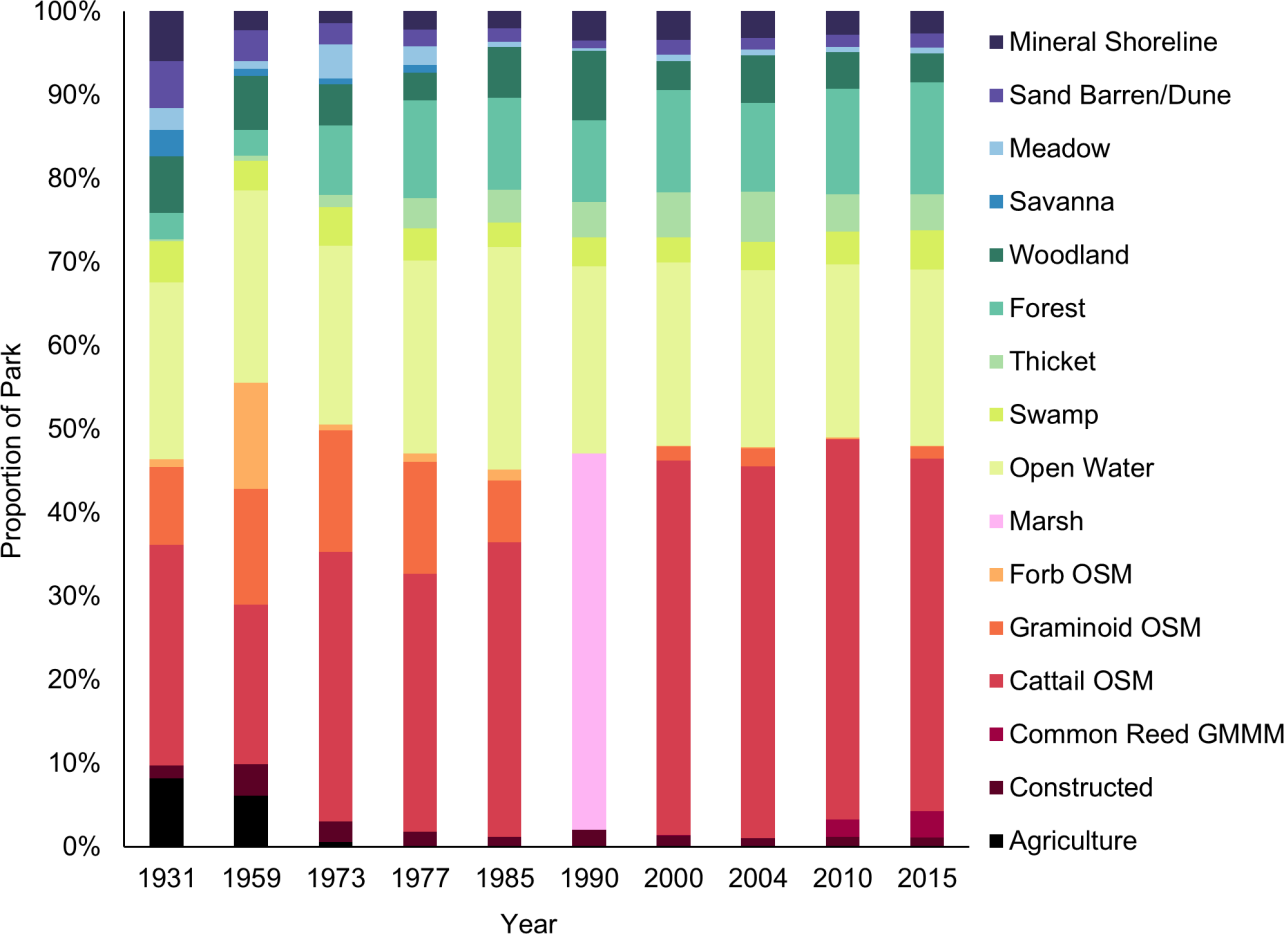
Habitat composition throughout the sampling period (1931 to 2015) in Point Pelee National Park. The quality of the 1990 image did not permit classification of marsh ecosites/vegetation types (i.e., common reed, cattail organic shallow marsh, graminoid organic shallow marsh, forb organic shallow marsh) and is only presented at the community level (marsh). Habitat types comprising < 0.5% of the park are not shown.

**Fig 2:**
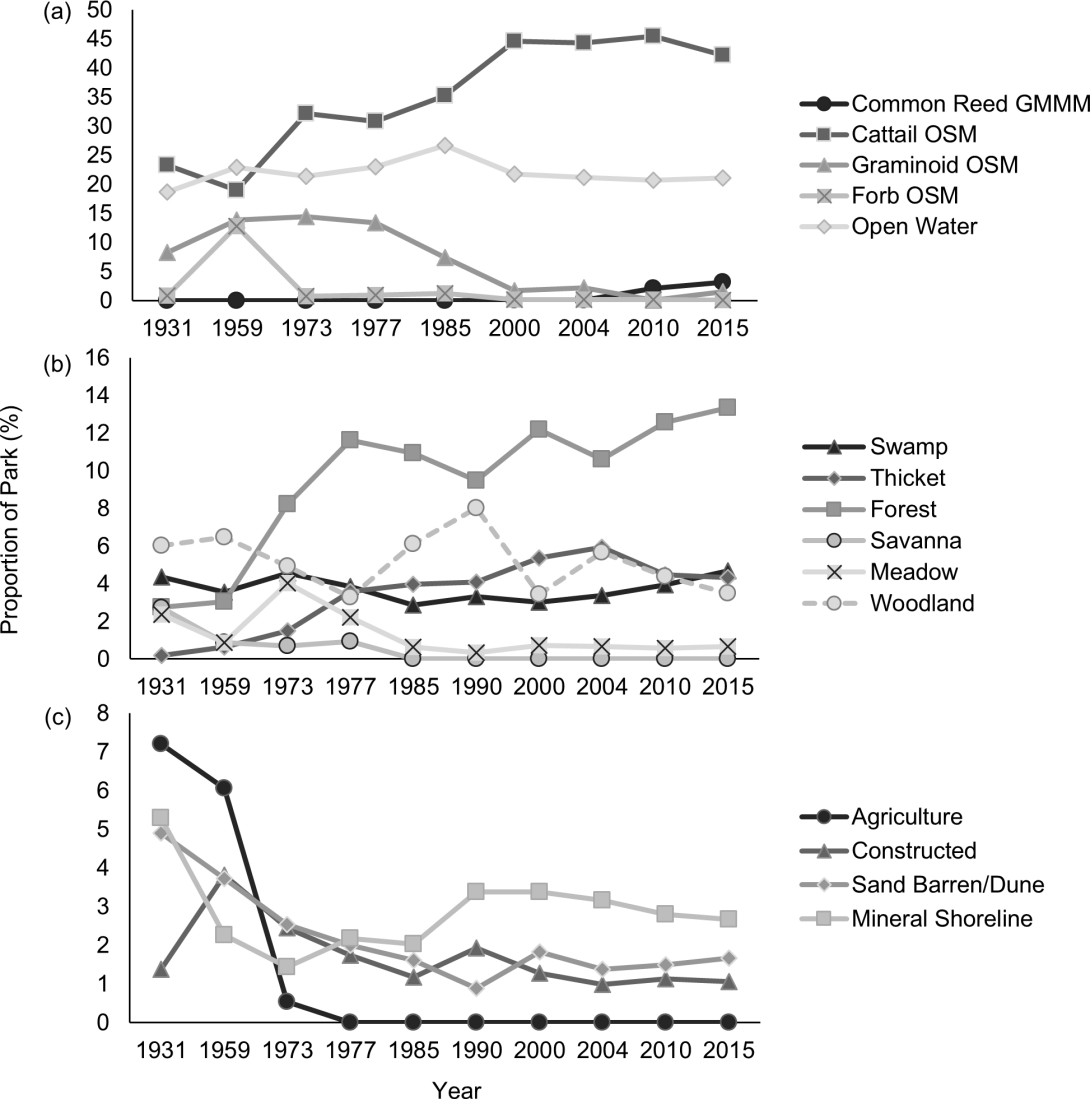
Land-cover trends over 85 years in Point Pelee National Park. (a) Marsh habitats, excluding 1990. (b) Upland habitats. (c) Shoreline, beach and anthropogenic classes. Habitat types comprising < 0.5% of the park are not shown.

Substantial changes in land cover proportions occurred in PPNP over the last 85 years (Fig 1), with the greatest occurring in the marsh (Fig 2A). Most notably, cattail organic marsh has doubled in total area from 309 ha to 625 ha (Fig 2A), while graminoid shallow marsh has decreased by 80% (S1 Table). Following a peak in 1959, the areal cover of forb shallow marsh has generally declined (Fig 2A). Although *Phragmites* had invaded the park prior to the end of the last century, the 2000 image was the earliest in which we could positively identify its presence. Over the course of the following 15 years, the number of *Phragmites* patches expanded from 4 to 166 unique patches, increasing to a density of 11 patches per 100 ha (0.27 patches in 1931). The amount of open water was relatively stable through time, comprising about 20% of the park’s total area. Although not to the same extent as that of the marsh, there were also changes in upland habitat classes (e.g., forest, thicket, savanna, and meadow classes; Fig 1); there was an increase in forest and thicket habitat types, while the amount of swamp remained fairly consistent (Fig 2B). Total forest area increased as mean patch area increased to 50 ha and patch extent reached 560 m (Table 3). Noticeable declines were observed for savanna and meadow habitats. About half of the core meadow habitat was lost between 1931 and 2015 (core area index; Table 3). The amount of sand dune habitat has remained consistent since 1990, but the proportion of treed and shrubby dune habitat significantly declined by 30 ha (R^2^ = 0.90, p < 0.0001). Active management within the park boundaries led to the decommissioning of all agricultural lands and reduced the number of buildings and roads since the 1930s (Fig 2C).

In 1931, the matrix (i.e., dominant habitat type in which patches are distributed throughout PPNP) was graminoid organic shallow marsh, with open water and forb organic shallow marsh interspersed. We detected a major change in the matrix that occurred between 1959 and 1973, when cattail organic shallow marsh became the dominant and most extensive feature and continued to take over the marsh until present-day conditions. Mean area for a cattail patch increased significantly from 64 ha to 336 ha, expanding at a rate of 4.12 ha per year (Table 3). The mean radius of gyration increased by 7.43 m per year, increasing from 377 m to almost a kilometer in extent. The core area of dense cattail habitat currently comprises 42% of the park landscape, with the largest patch totaling 28% of the cattail area. Furthermore, when we divided the landscape into patches of equal sizes, the amount of cattail increased from 13 ha to 142 ha (Table 3). In contrast to cattail habitat, percentage of available forb core area has been completely lost from the park (declined to 0% from 50%; Table 3), leaving the remaining 1.2 ha as edge habitat. Although the amount of open water remained consistent through time (Fig 2A), the percentage of available core area has increased to 83%, but the number of disjunct areas has declined from 79 to 33 patches, indicating an overall reduction in aquatic connectivity (Table 3).

**Fig 3:**
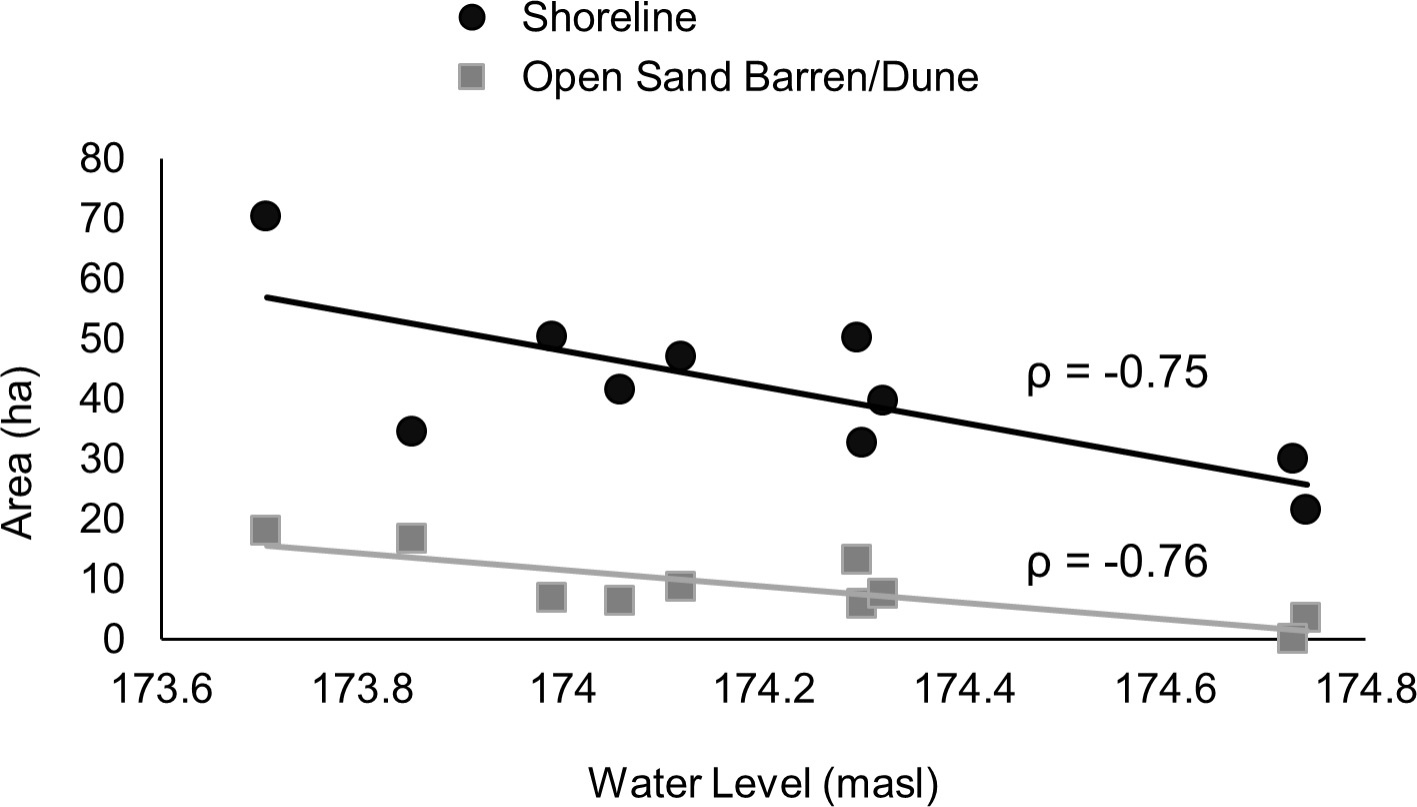
Plot of areal extent of Point Pelee National Park and open sand barren/dune as a function of annual mean water level in Lake Erie (meters above sea level). Both areal extent of Point Pelee National Park and open sand barren/dune were significantly correlated with water level (*p* = 0.01); Spearman’s rho correlation coefficient is shown separately for each correlation.

**Fig 4:**
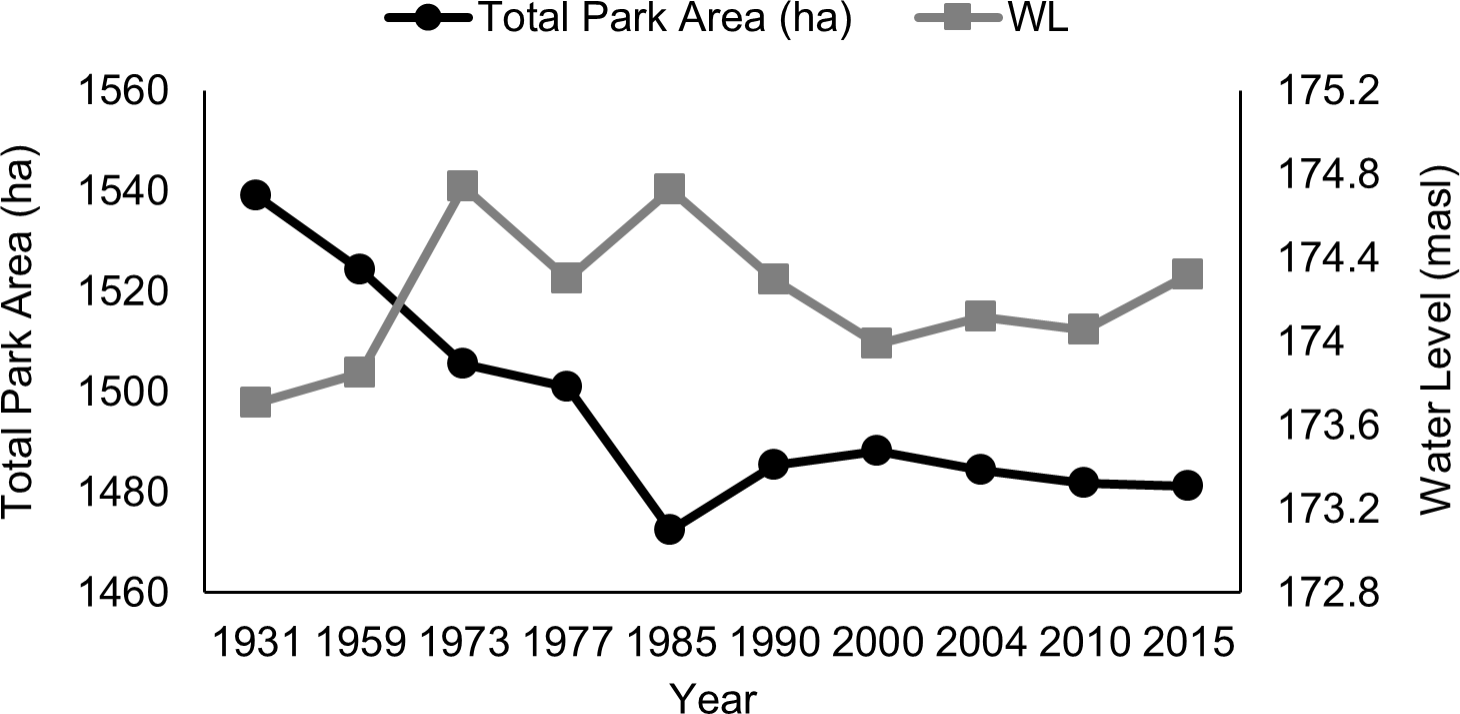
Total area of Point Pelee National Park from 1931 to 2015. A net loss of 58 ha was experienced over this time period. Years with a larger park area tend to be associated with higher water levels and vice versa.

Point Pelee is a sand spit and is constantly subjected to erosional and depositional forces. Throughout 1931 to 2015, erosional forces have been greater than depositional forces along the eastern shorelines. Although the western shoreline has seen some deposition, there was a net loss in shoreline area totaling 72 ha. Total length of the shoreline has also decreased from 19.2 km to 18.0 km. The area of mineral shoreline and sand barrens/dunes has been declining through time, with some observed fluctuations (Fig 2C; S1 Table). We found a significant negative correlation between water level of Lake Erie and the amount of shoreline (-0.75, *p* = 0.01) and open sand barren/dune habitat (-0.76, *p* = 0.01; Fig 3) in PPNP; such a significant correlation with water levels was not identified for any other habitat class. There was also a decline in total park area over time, which appeared to be associated with Lake Erie water levels (Fig 4). Although total park area tended to decline as water levels rose (1931–1985), it did not increase when lower water levels returned between 1990 to 2015 (Fig 4). Consequently, PPNP has experienced an overall net loss of 58 ha since 1931.

### Change-detection

Our change-detection analysis revealed that over 650 ha, 43–44% of the total park area, underwent a change in habitat type between 1959 and 2015. Most notably, loss of forb and graminoid marsh was succeeded by 139 ha and 188 ha of cattail marsh, respectively. Over 50 ha of open water was infilled by cattail marsh. As a result of management actions, approximately 89 ha of constructed and agricultural land was reclaimed as forest or thicket. The increase in amount of forest was also because of succession of woodland areas (72 ha).

**Fig 5:**
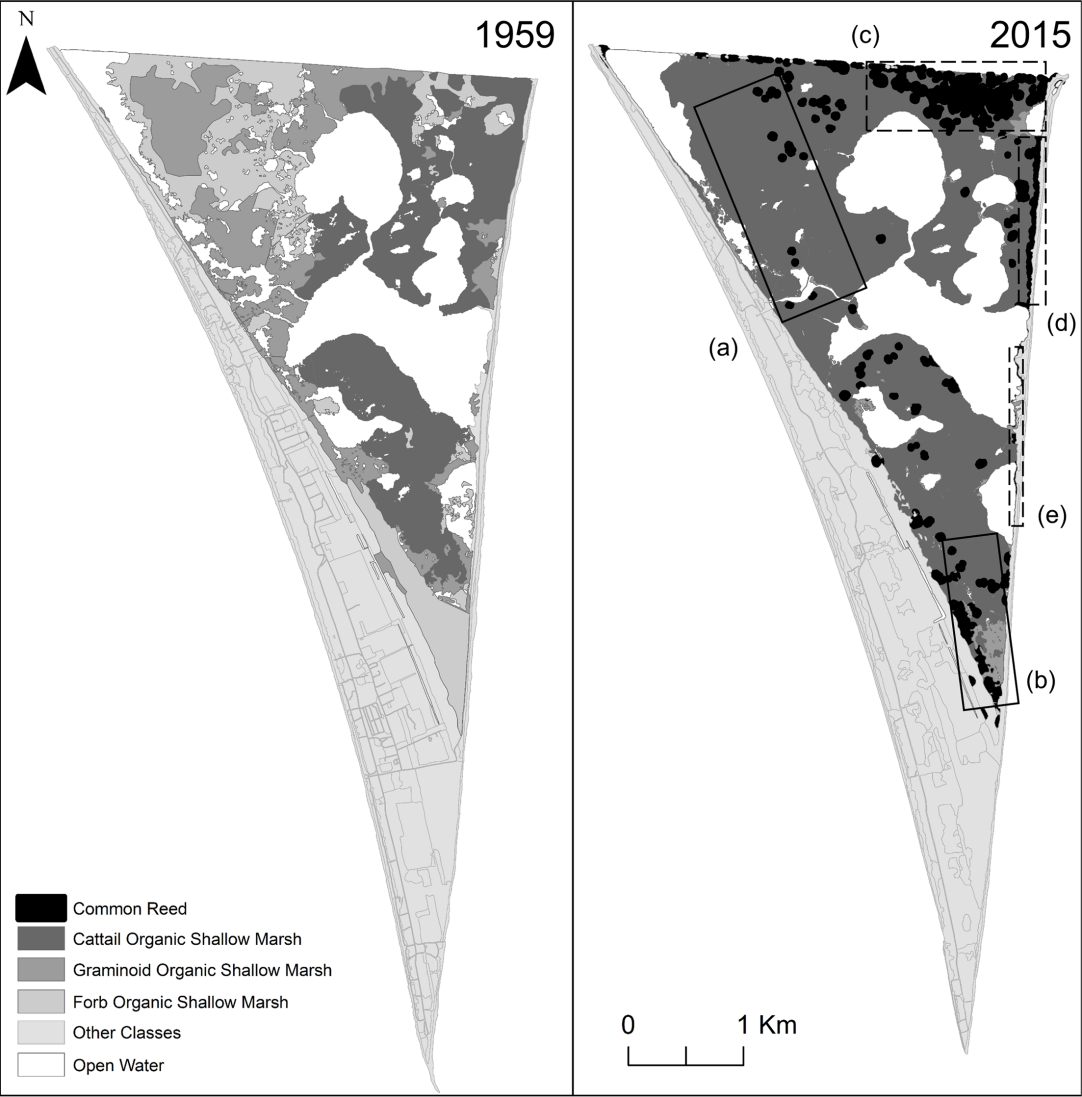
Change in marsh habitat in Point Pelee National Park between 1959 and 2015. (a) and (b) indicate hotspots of major habitat change and infilling within the marsh (solid-lined boxes). (c), (d), and (e) indicate hotspots of invasive common reed (dashed-lined boxes).

We identified five hotspots within the park that experienced major changes (Fig 5). There are two hotspots in the marsh where habitat transitioned from a diverse mixture of broad- and narrow-leaved emergent vegetation types interspersed with pools of water in 1959 to almost being completely infilled by dense cattails in 2015 (Hotspot A and B; Fig 5A and 5B). In 2015, over 90% of the *Phragmites* distribution had displaced marsh habitats (i.e., cattail, forb, graminoid OSM), while the remaining 10% occupied open water, swamps, and sand dunes. Concurrent with the spread of *Phragmites* throughout the park, large homogeneous stands infiltrated marsh habitat towards the northern end (Hotspot C; Fig 5C); long stretches of *Phragmites* also became established along majority of the eastern beach, creating a barrier between the beach and marsh (Hotspot D and E; Fig 4D and 4E).

## Discussion

### Long-term habitat changes

Consistent with our expectations, we confirmed that long-term changes in habitat availability and distribution have occurred in Point Pelee National Park. First, both the diversity of marsh habitat and connectivity of aquatic habitat patches have been reduced over the past 85 years, similar to changes noted in other Ontario wetlands [39]. Between 1959 and 1973, the marsh matrix transitioned from a graminoid organic shallow marsh interspersed with patches of open water and forb organic shallow marsh, to a cattail-dominated marsh with very few patches of open water. Such major changes in habitat distribution and isolation has implications for herpetofauna and their ability to access required habitat types during the active season. For instance, in 1931, there had been abundant shallow aquatic habitat that would have provided important foraging, mating, and overwintering habitat for herpetofauna [18,21]. By 1973, however, the dispersed shallow pools had coalesced into large deep pools and had become surrounded by large dense stands of cattails. In such a habitat matrix, herpetofauna might have been forced to travel up to a kilometer through dense cattail stands to access critical foraging and nesting habitat, such as graminoid and forb shallow marsh, mixed meadow marsh, and sand barrens and dunes. Since patches throughout the landscape continued to expand at a rate of 1.84 ha per year, unfavourable habitat patches (e.g., *Phragmites*) would have become increasingly difficult to traverse, while critical habitats would have become more isolated and disconnected.

High-quality thermoregulation habitat likely became a limiting factor for herpetofauna in PPNP. For turtles, thermoregulation opportunities may have become severely reduced as dense, homogenous cattail beds formed, leaving almost no open water [24]. For snakes, loss of open terrestrial habitats such as savannas and meadows may have reduced important thermoregulation habitats. Open terrestrial habitats have been found to provide a variety of environmental temperatures, and by basking or seeking shelter, snakes can control their body temperature [40]. Moreover, the decline in treed and shrubby sand dunes reduces availability of cover objects known to be important for certain snakes [27]. In a similar thermal environment to PPNP, snakes near Ottawa, Ontario, preferred open habitats over forest likely because it enabled behavioural thermoregulation [40]. The lower thermal quality of forests compared to open habitats resulted in snakes preferring open habitat at both the microhabitat and macrohabitat scale [41]. In PPNP, there had been a succession from woodland habitat with open canopy to forests with closed canopies, as well as a decline in savanna and meadow that resulted in a total loss of 150 ha of open habitat; such a change could have resulted in reduced thermoregulatory opportunities [42–44]. Furthermore, loss of large patches of early successional habitats has been identified as a factor contributing to declines in snake species richness [45].

The third major habitat change in PPNP was the invasion of *Phragmites australis* into marsh and beach habitats, posing a significant threat to the quality and amount of potential breeding and nesting habitat for herpetofauna. Growth of *Phragmites* along the eastern beach has created a potential barrier for species, particularly turtles, from moving between the marsh and beach during the nesting season. In addition, growth of *Phragmites* in known turtle nesting beaches can lower nest temperatures through shading [46]. In a similar sand dune and shallow marsh ecosystem, the Fowler’s toad (*Anaxyrus fowleri*) experienced population declines following loss of breeding habitat to *Phragmites*, even though there had been minimal loss of adult habitat [47]. Although *Phragmites* was only at image-detectable levels in 2000, it had invaded PPNP sometime between 1970 and 1990 [48], and continues to proliferate.

Legislation can protect a parcel of land from being altered by human activities, but it cannot protect the parcel from changes in hydrological variability nor natural successional processes that can affect marsh habitat and impact species that are reliant on a diverse, interconnected system (e.g., [49]). Moreover, habitat availability and suitability are expected to evolve with climate change in light of predicted increases in temperature and decreases in precipitation [50]. As both natural and anthropogenically accelerated forces alter sensitive marsh habitats, active management towards a more diverse vegetative and structural state will provide a range of opportunities for feeding, breeding, overwintering, and thermoregulating, and likely benefit the highest number of native and rare species.

### Habitat management and restoration

We identified five hotspots based on areas that have undergone the greatest amount of habitat change. The first two hotspots were once characterized by a mix of shallow water wetlands (cattail, graminoid, forb, open water) but have been completely succeeded by cattails. Efforts to increase the number of open-water patches within cattail beds could provide movement corridors and additional basking opportunities for reptiles; however, management strategies must be carefully considered from a herpetological perspective to ensure no damage is done to occupied habitats and individuals are not harmed.

We identified the next three hotspots as areas where *Phragmites* has either formed a barrier wall between the marsh and the beach or formed large, dense homogeneous patches. The control of *Phragmites* is important to recovering beach nesting habitat and marsh habitat. In particular, the barrier of *Phragmites* between the beach and marsh is concerning for turtles in search of nesting habitat. Resident wetlands and nesting sites must be closely linked and the connecting corridor must provide passable habitat [51], which is likely not the case along the eastern beach. If *Phragmites* continues to expand into sandy habitats, high water level years can compress the amount of available nesting habitat since sand barren and dune habitats are correlated with changes in Lake Erie water levels. Since 1931, we have identified a net loss of 58 ha of park area because of erosion, and the beach is vulnerable to further losses. Removal of *Phragmites* along the beach is key in restoring connectivity to the marsh and maintaining high quality nesting habitat.

In addition to the changes and losses in critical habitat we identified between 1931–2015, there are additional stressors that may have directly or indirectly contributed to species declines in PPNP including the historical loss of 2000 ha of marsh that existed beyond the current park boundary [23], disturbances from high volumes of visitors [52], increases in mesopredator species that predate on turtle eggs [23], historic use of chemicals [53], and the geographic isolation of the park reducing immigration and rescue effects [8, 23].

## Conclusion

Without actions to recover lost and degraded habitat herpetofauna species will continue to lose critical habitat. Long-term persistence of herpetofauna is dependent upon access to critical habitats for mating, foraging, nesting, and overwintering. We have provided several lines of evidence to show how critical habitat has become lost and/or fragmented in Point Pelee over the course of 85 years, even though this social-ecological-system had been protected as a national park. We acknowledge that many factors contribute to the extirpation or decline of a species; however, loss, fragmentation and degradation of habitat within the park boundary has undoubtedly played a role in the decline of herpetofauna in PPNP, given that these are the primary causes of species declines worldwide. Therefore, future management plans should include restoring some of the original habitat features identified in this study and future research should evaluate habitat management strategies from a herpetological perspective to determine best practices. A time-sensitive step includes control of invasive *Phragmites australis* to prevent further encroachment on critical reproductive and thermoregulatory habitat. Habitat restoration alone will not be enough to recover species at-risk but evaluation of historical habitat and long-term changes is the cornerstone to assessing restoration needs and designing an effective management approach. The creation of suitable habitat and assessment of long-term changes can help support the incredible floral and faunal diversity of Point Pelee National Park and contribute to a comprehensive feasibility study for re-establishing lost herpetofauna [28].

## Supporting information

S1 Table Landscape composition in hectares in Point Pelee National Park from 1931 to 2015. Habitat types are presented according to the greatest amount of change between 1931 and 2015. The quality of the 1990 image prevented classification beyond the marsh community class and therefore we have no data (nd) for ecosites/vegetation types.
(DOCX)
