# Supplementary material for: Long-term habitat changes in a protected area: Implications for herpetofauna habitat management and restoration
Source: PLoS One. 2018 Feb 14;13(2):e0192134. doi: 10.1371/journal.pone.0192134 (PMC5812606; doi:10.1371/journal.pone.0192134)
Supplement: S1 Table — Habitat types are presented according to the greatest amount of change between 1931 and 2015. The quality of the 1990 image prevented classification beyond the marsh community class and therefore we have no data (nd) for ecosites/vegetation types. (DOCX) [file pone.0192134.s001.docx]

| Habitat | 1931 | 1959 | 1973 | 1977 | 1985 | 1990 | 2000 | 2004 | 2010 | 2015 | Change in landcover 1931-2015 (ha) |
| --- | --- | --- | --- | --- | --- | --- | --- | --- | --- | --- | --- |
| Cattail Organic Shallow Marsh | 309.0 | 289.5 | 484.6 | 461.9 | 519.4 | nd | 663.6 | 656.9 | 673.0 | 624.9 | +315.9 |
| Forest | 36.5 | 46.4 | 124.1 | 174.6 | 161.1 | 140.7 | 181.2 | 157.4 | 186.5 | 197.9 | +161.4 |
| Open Water | 247.8 | 349.3 | 321.3 | 345.4 | 392.9 | 322.1 | 324.2 | 313.7 | 306.2 | 312.0 | +64.2 |
| Thicket | 2.7 | 9.8 | 22.7 | 53.6 | 58.5 | 60.9 | 80.0 | 88.3 | 66.3 | 64.2 | +61.5 |
| Common Reed | 0.0 | 0.0 | 0.0 | 0.0 | 0.0 | nd | 1.1 | 0.6 | 31.4 | 47.3 | +47.3 |
| Swamp | 58.0 | 54.1 | 68.8 | 57.7 | 42.4 | 49.5 | 45.0 | 50.0 | 58.3 | 69.4 | +11.4 |
| Graminoid Mineral Meadow Marsh | 0.0 | 2.7 | 3.8 | 5.7 | 0.0 | nd | 6.8 | 6.9 | 3.3 | 4.3 | +4.3 |
| Mixed Mineral Meadow Marsh | 0.0 | 0.0 | 1.2 | 0.8 | 0.0 | nd | 0.7 | 0.3 | 0.2 | 0.2 | +0.2 |
| Agriculture | 95.7 | 92.3 | 8.2 | 0.0 | 0.0 | 0.0 | 0.0 | 0.0 | 0.0 | 0.0 | -95.7 |
| Graminoid Organic Shallow Marsh | 110.0 | 210.8 | 217.8 | 200.4 | 109.4 | nd | 25.0 | 31.8 | 1.0 | 21.8 | -88.2 |
| Sand Barren/Dune | 65.1 | 56.8 | 38.2 | 30.2 | 65.6 | 41.9 | 27.2 | 20.6 | 22.3 | 24.7 | -40.4 |
| Savanna | 36.2 | 13.3 | 10.7 | 14.0 | 0.0 | 0.0 | 0.0 | 0.0 | 0.0 | 0.0 | -36.2 |
| Mineral Shoreline | 70.4 | 34.4 | 21.5 | 32.7 | 30.0 | 50.2 | 50.3 | 47.0 | 41.5 | 39.6 | -30.8 |
| Woodland | 80.2 | 98.8 | 74.2 | 49.6 | 90.1 | 119.0 | 51.1 | 84.1 | 64.8 | 51.9 | -28.3 |
| Meadow | 31.5 | 13.2 | 60.7 | 33.4 | 9.3 | 4.9 | 10.9 | 10.0 | 8.5 | 10.1 | -21.4 |
| Forb Organic Shallow Marsh | 10.7 | 194.5 | 10.6 | 14.7 | 18.7 | nd | 2.0 | 2.0 | 1.7 | 1.2 | -9.5 |
| Constructed | 18.4 | 58.2 | 37 | 26.1 | 17.2 | 28.7 | 19.1 | 14.7 | 16.8 | 15.7 | -2.7 |
|  |  |  |  |  |  |  |  |  |  |  |  |

**S1 Table. Landscape composition in hectares in Point Pelee National Park from 1931 to 2015.**

Habitat types are presented according to the greatest amount of change between 1931 and 2015. The quality of the 1990 image prevented classification beyond the marsh community class and therefore we have no data (nd) for ecosites/vegetation types.
